# New Polymer Composites with Aluminum Phosphates as Hybrid Flame Retardants

**DOI:** 10.3390/ma16010426

**Published:** 2023-01-02

**Authors:** Kamil Dziuba, Krystyna Wnuczek, Patryk Wojtachnio, Rodolphe Sonnier, Beata Podkościelna

**Affiliations:** 1 Department of Organic Chemistry, Faculty of Chemistry, Institute of Chemical Sciences, Maria Curie Skłodowska University, Gliniana 33, 20-614 Lublin, Poland; 2 Department of Polymer Chemistry, Faculty of Chemistry, Institute of Chemical Sciences, Maria Curie Skłodowska University, Gliniana 33, 20-614 Lublin, Poland; 3 Polymers, Composites and Hybrids (PCH), IMT Mines Alès, 6 Avenue de Clavières, 30100 Alès, France

**Keywords:** aluminum hydroxide, epoxy resin, aluminum–organophosphorus hybrid, thermal properties, flammability tests

## Abstract

Polymeric aluminum organophosphates are a class of nanostructured aluminum-based compounds that can be considered organic and inorganic hybrid materials. Aluminum phosphates have attracted considerable interest due to their ability to enhance composite materials’ mechanical characteristics, lightweight, and thermal properties. Extensive studies have shown the potential of aluminum organophosphates as a component in the development of fire-retardant materials. Aluminum–organophosphorus hybrid (APH) materials have been prepared by reacting aluminum oxide hydroxide (boehmite) with alkyl and aryl phosphoric acids and used to prepare composites with epoxy resin. Boehmite is an aluminum oxide hydroxide (γ-AlO(OH)) mineral, a component of the aluminum ore bauxite. In this work, the composites based on epoxy resin Epidian 601 and commercial curing agent IDA were obtained. Pure boehmite and APH hybrids were added as flame retardants. FTIR and TGA analysis showed that obtained APH possesses a hybrid structure, high thermostability, and various morphologies. These new APH were incorporated into epoxy resin. The infrared spectroscopy confirmed the structure of hybrids and composites. Pyrolysis combustion flow calorimetry (PCFC) and cone calorimeter analyses were performed to assess the flame retardant properties of the composites. The results showed that the incorporation of 17 wt% APH allows a reduction of heat release rate but to a limited extent in comparison to pure boehmite, which is due to the different decomposition mechanisms of both boehmite and hybrids. The cone calorimetry test showed that residue contents correspond quite well to the mineral fraction from boehmite only. The hybrid APHs appear no more efficient than pure boehmite because the mineral fraction in APH is reduced while phosphate fraction cannot promote significant charring.

## 1. Introduction

Technological and scientific advances in the 21st century had already made polymer-based materials the first choice for a huge range of applications; these materials are quickly replacing others, and they dominate every aspect of daily life. In recent decades, composite materials have overtaken aluminum and steel alloys in a variety of applications [1,2]. Materials include organic polymeric frameworks reinforced with glass, aramid, carbon, or natural fibers and provide unique physicochemical, thermal, chemical, and mechanical capabilities while retaining low density, high specific stiffness and strength, corrosion resistance, strong fatigue endurance, and low thermal expansion [3]. As a consequence of the organic origin of the polymeric matrix and fiber, composite materials have low fire resistance. Similar to other thermosetting resins, the organic matrix of the crosslinked epoxy laminate decomposes in high temperatures (300–400 °C), emitting heat, smoke, soot, as well as harmful fumes [4]. During the combustion of a polymer, the condensed phase typically involves four specific processes. Different functional groups (or atoms) that are not part of the polymer matrix may be removed at the same time. This reaction is referred to as chain stripping. Cross-linking of the various radicals created during chain scission to build more or less stable char is the final dominating reaction. Regarding epoxy resins, a initial phase of heat decomposition is the dehydration of the secondary alcohol, formed by the crosslinking reaction to produce allylic amides [5]. The non-saturated part of the molecule can then undergo isomerization and allylic-oxygen bond cleavage [6]. For amine crosslinkers, the weak C-N link produced while curing will then undergo allylic-nitrogen bond breaking, resulting in the formation of volatile particles or contributing to charring [4].

However, a flame retardant can be introduced to enhance the flame retardancy of the crosslinked resin [7,8]. Consequently, very important is to recognize the fact that each application requires a unique composition (e.g., resins, hardeners, or fire retardants). According to the fire loop, there are various points at which a fire can be suppressed. The flame retardant can operate in the gaseous phase by inhibiting the exothermic reaction (oxidation) in the flame by radical scavenging, hence limiting the energy feed-back to the polymer [9]. A flame retardant can also create a thermal barrier (charring) on the surface of the condensed phase, which inhibits heat transfer back to the burning polymer [10]. Increased charring formation reduces the number of flammable gases reaching the flame, resulting in its extinguishment. Because they catalyze the formation of char, flame retardants that employ the second mechanism is known as condensed phase active. Until recently, the predominance of flame retardants was halogen-based, with tetrabromobisphenol A (TBBPA) being the most commonly used flame retardant in epoxy resins. Bromine-derived retardants (such as TBBPA) are well known to behave like flame poisons when exposed to thermal stress by producing volatile bromine radicals [11]. This leads to an interruption in the fire loop.

Thus, halogen flame retardants dominate the industry for flame retardants used in electrical and electronic applications. To conform to new environmental standards, however, chlorinated and particularly brominated substances are being phased out (such as REACH, WEEE, and RoHS). According to REACH, only registered flame retardant chemicals with hazard information can be utilized. As a response, a rising number of laboratories and businesses have begun to incorporate alternative flame retardants into their product lines to comply with these new laws and offer products that are considerate of human health and the environment.

In electrical and electronic applications, alternatives to halogen flame retardants could be classified into three main categories: inorganic flame retardants, nitrogen-based flame retardants, and phosphorus-based flame retardants. As a flame retardant, metal hydroxides such as aluminum hydroxide (ATH) and magnesium hydroxide (MDH) have various beneficial effects. They are inexpensive, readily accessible, non-toxic, and environmentally beneficial. However, extremely high loadings (up to 60%) are necessary to achieve flame retardancy. Of that kind, high loadings have detrimental effects on the final product’s properties. Aluminum-oxide-hydroxide (boehmite) possesses a significantly higher thermal stability and can be utilized in epoxy systems undergoing lead-free soldering. As stated previously, lead-free soldering needed higher processing temperatures, requiring additives with greater thermal stability. Current PCB (Printing Circuit Board) systems consisting of a novolac epoxy resin and DICY (dicyandiamide) as a hardener must withstand 288 °C without delamination [12]. Metal hydroxides are frequently utilized as synergists with more recent phosphorus-based flame retardants (e.g., metal phosphinates) [13].

The majority of the study on halogen-free flame retardants focuses on phosphorus-based solutions, which are predicted to have the largest increase in market share. Phosphorus flame retardants (organic and inorganic) are generally considered non-hazardous and do not generate toxic gases because phosphorus is predominantly bound to the char [14,15]. Organophosphorus compounds offer superior physical qualities and lower loading than conventional fillers (e.g., ATH). However, these are still more expensive than commonly used flame retardants (e.g., ATH or TBBPA), therefore their widespread use is still in the development process. Nonetheless, the industrial production of phosphorus flame retardants will contribute to their price decline. Although industry and academic efforts resulted in various phosphorus-based flame retardant solutions, empirical research accounted for the majority of the achievements. Considering that the decomposition of an epoxy resin is highly dependent on the type of resin and the hardener (and other additives) used, there is no common flame retardancy mechanism for phosphorus compounds. Phosphorus compounds that promote hydrogen recombination and molecular phosphorus absorption of hydroxyl radicals are also classified as gas phase active. Under thermal stress, other phosphorus-based flame retardants tend to create polyphosphoric acids, thus promoting the formation of thermally stable polymers (charring). A significant advantage of phosphorus flame retardants is that both processes may be active [16]. Through chemical customizing, it is feasible to favor one or the other pathway.

There are two approaches to making flame-retardant polymer. The flame retardant is either incorporated as an additive (henceforth referred to as non-reactive FR) or covalently bonded to the polymer (henceforth referred to as reactive FR). Both reactive and non-reactive flame retardants have significant advantages in a wide range of applications. The largest market share in flame-retardant polymers is occupied by additives. To achieve the desired result, substantial loadings (up to 60 wt%) are required, which frequently have a detrimental influence on the material and mechanical properties of the polymer [17]. Since the late 1970s, additives such as metal salts of dialkyl phosphinates have been recognized as efficient flame retardants [18]. As a flame retardant, Ticona studied a broad variety of zinc, aluminum, and calcium salts of dialkyl phosphinates [19]. Aluminum alkyl phosphinates, which were originally developed for polyamides and fiberglass-reinforced polyesters, provide a significant FR effect with the addition of ~40 wt%. Aluminum salts of diethyl phosphinate were developed by Clariant and deployed widely in commercial use [20,21]. Methyl phosphonic acid [22,23] and phosphoric acid monoesters [24,25,26,27,28,29] have been reported as useful reagents for the synthesis of organically modified microporous aluminum phosphonates, aluminum phosphate clusters, and mesostructured lamellar aluminum phosphates consisting of AlPO_4_ layers separated by covalently bonded long alkyl chains. Recent studies have shown that the reactivity of boehmite with triethyl phosphate or diphenyl phosphoric acid (DPPA) in boiling xylene leads to the formation of aluminum phosphates which was attributed to a closely packed hexagonal structure consisting of catena-Al(DPPA)_3_ polymer chains [30,31,32].

In this paper, we report the synthesis and characterization of three populations of aluminum phosphate-hybrid materials that are formed by reacting phosphate acids in the presence of boehmite as well as some preliminary investigations on the efficiency of these materials as flame retardants in polymer composites. ATR/FT-IR analyses were also carried out to confirm the structure of the hybrids. Polymer composites were prepared by mixing the epoxy resin (Epidian 601) and curing agent (IDA) to create a macroscopically homogeneous material. Our aluminum phosphate-hybrid materials and pure boehmite were added to the resin to check their influence on flame retardancy measured by pyrolysis-combustion flow calorimetry and cone calorimetry. The pure sample of EP601 + IDA was prepared as reference material.

## 2. Materials and Methods

### 2.1. Materials

The epoxy resin Epidian^®^ 601 (EP601) as well as curing agent IDA were purchased from CIECH Resin Sp.z.o.o. (Nowa Sarzyna, Poland). Epidian^®^ 601 is a clear, viscous liquid of light color, epoxy number: 0.500–0.550 mol/100 g, viscosity at 25 °C: 700–1100 mPas, density at 20 °C: 1.140–1.170 g/cm^3^. The curing agent IDA contains 4,4′-isopropylidenedophenol, an oligomeric reaction product with 1-chloro-2,3-epoxypropane, a reaction product with 3-aminomethyl-3,5,5-trimethylcyclohexylamine, isophorone diamine. Organophosphorus reagents: diethyl chlorophosphate, diphenyl chlorophosphite, and bis(2-ethylhexyl)phosphate was purchased from Sigma-Aldrich (Darmstadt, Germany). The high-purity aluminium oxide hydroxide (boehmite) PURAL SB was made available for our research courtesy of the company Sasol Germany GmbH. Tetrahydrofuran (THF), mesitylene, diethyl ether (Et_2_O), and chloroform (CHCl_3_) were purchased from Sigma-Aldrich also. The sodium base, hydrochloric acid, and anhydrous magnesium sulfate come from the company Merck (Darmstadt, Germany). The listed materials were used as received without further purification.

### 2.2. Methods

The NMR spectra have been registered on a Bruker AV500 (^1^H 500 MHz, ^31^P 202 MHz, ^13^C NMR 126 MHz) spectrometer. All spectra were received in deuterated chloroform solutions unless mentioned otherwise, and the chemical shifts (d) are expressed in ppm using the internal reference to TMS (tetramethylsilane) with the solvent as an internal indicator and the external reference to 85% H_3_PO_4_ in D_2_O for ^31^P. The coupling constants (J) are given in Hz. The abbreviations of signal patterns are as follows: s, singlet; d, doublet; t, triplet; q, quartet; m, multiplet; b, broad.

The attenuated total reflection (ATR) have been registered using infrared Fourier transform spectroscopy (ATR-FT/IR) on a TENSOR 27, Bruker spectrometer, equipped with a diamond crystal (Germany). The spectra in the range of 600–4000 cm^−1^ with 64 scans per spectrum at a resolution of 4 cm^−1^ were recorded.

The shape analysis of the particles used in the studied composites was carried out by the optical microscope (Morphologi G3, Malvern, UK).

Thermal analysis was performed by using STA 449 Jupiter F1, Netzsch (Selb, Germany). Samples were heated in the range of 30–800 °C at a rate of 10 °C·min^−1^ in a dynamic atmosphere of helium (20 cm^3^·min^−1^). The S TG-DSC type sensor thermocouple with an empty Al_2_O_3_ crucible as a reference was applied.

Flammability at the microscale was recorded using a pyrolysis combustion flow calorimeter (PCFC, Fire Testing Technology, East Grinstead, UK), developed by Lyon and Walters (Lyon & Walters, 2004). A fixed amount of sample (i.e., 2–3 mg) was heated at a rate of 1 °C·s^−1^ under a nitrogen atmosphere (flow: 100 mL·min^−1^) from 150 to 750 °C. The products of the degradation were transferred to a combustion chamber where they are mixed with an excess of oxygen at 900 °C (complete combustion). The heat release rate is measured using Huggett’s relation [33] according to the oxygen depletion method.

The flammability was also investigated using a cone calorimeter (Fire Testing Technology, East Grinstead, UK) according to the ISO 5660 standard. A horizontal sample sheet of 90 × 60 mm^2^ was placed 2.5 cm below a conic heater. The thickness of all samples was 2 mm. Typically, samples were exposed to 35 kW·m^−2^ heat flux in well-ventilated conditions in the presence of a spark igniter to force the ignition. The edges as well as the bottom of the samples were covered by aluminum foil as usual. The heat release rate was calculated also from Huggett’s relation, as in PCFC.

The hardness of the materials was measured by the Shore D method using a 7206/H04 analog hardness testing apparatus, Zwick (Ulm, Germany). The reading was taken after 15 s.

## 3. Synthesis of the Phosphates and Aluminum Phosphates

### 3.1. Procedure for the Gram-Scale Synthesis of Diethyl Phosphate (DEPA)

Diethyl chlorophosphate (35 g, 0.2 mol) was dissolved in THF (250 mL) and cooled to 0 °C. Then, 200 mL of NaOH (1 M, in water) was added dropwise into the solution and stirred for 30 min at 0 °C. The reaction mixture was allowed at room temperature for one hour and evaporated under reduced pressure. The resulting precipitate was washed with Et_2_O (3 × 100 mL) and filtered. The solvent was evaporated and the remaining liquid was acidified with HCl (2 M) solution to pH = 3–4 and extracted 4 × 50 mL of CHCl_3_. The combined organic layers were dried with MgSO_4_, filtered, and evaporated under vacuum to yield 27.1 g (87%) of diethyl phosphate.

^1^H NMR (500 MHz, Chloroform-d) δ 11.58 (s, 1H), 4.10 (qdd, J = 7.2, 4.5, 2.8 Hz, 2H), 1.37–1.30 (m, 3H); ^13^C NMR (126 MHz, Chloroform-d) δ 63.65, 15.98 (d, J = 7.1 Hz); ^31^P NMR (202 MHz, Chloroform-d) δ 0.14.

### 3.2. Procedure for the Gram-Scale Synthesis of Diphenyl Phosphate (DPPA)

Diphenyl chlorophosphate (52.7 g, 0.2 mol) was dissolved in THF (250 mL) and cooled to 0 °C. Then, 200 mL of NaOH (1 M, in water) was added dropwise into the solution and stirred for 30 min at 0 °C. The reaction mixture was allowed at room temperature for one hour and evaporated under reduced pressure. The resulting precipitate was washed with Et_2_O (3 × 100 mL) and filtered. The solvent was evaporated and the remaining liquid was acidified with HCl (2 M) solution to pH = 3–4 and extracted 4 × 50 mL of CHCl_3_. The combined organic layers were dried with MgSO_4_, filtered, and evaporated under vacuum to yield 49 g (98%) of diphenyl phosphate.

^1^H NMR (500 MHz, Chloroform-*d*) δ 12.00 (s, 1H), 7.33 (dd, *J* = 8.7, 7.2 Hz, 4H), 7.24–7.17 (m, 6H); ^13^C NMR (126 MHz, Chloroform-*d*) δ 150.46 (d, *J* = 7.1 Hz), 129.75, 125.39, 120.23; ^31^P NMR (202 MHz, Chloroform-*d*) δ −10.82.

**Figure 1:**
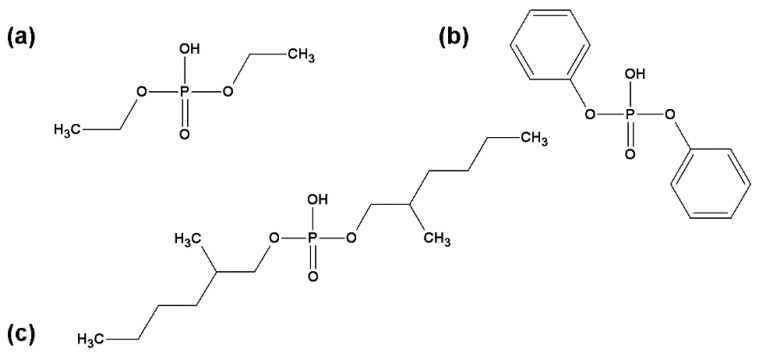
Structure of phosphates: (**a**) diethyl phosphate (DEPA—20 wt% of phosphorus), (**b**) diphenyl phosphate (DPPA—12.4 wt% of phosphorus), (**c**) bis(2-ethylhexyl)phosphate (DEHPA—10.5 wt% of phosphorus).

Figure 1 shows the structures of phosphates.

### 3.3. Modifications of Boehmite with Phosphates

Boehmite (15 g, 0.25 mol) and phosphates (0.75 mol) were refluxed in a Soxhlet apparatus with 500 mL of mesitylene for 48 h. The resulting slurry was decanted and then centrifuged to yield a white solid material of aluminium phosphates, which was dried in a vacuum at 110 °C.

## 4. Composites Preparation

**Figure 2:**
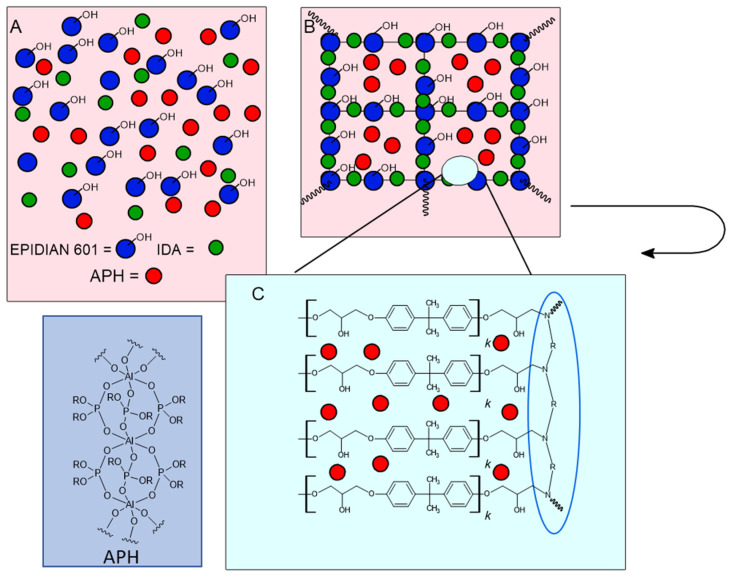
Proposed scheme of polymeric composites structure: (**A**) before and (**B**) after polymerization reaction, (**C**) presents the structure of bond between EP601 and IDA.

An appropriate amount of epoxy resin EP601 was added to the curing agent IDA. The chemicals were stirred at room temperature until a homogenous solution was obtained. Then the boehmite or aluminum phosphates were added in the amount of 17 wt%. The whole content was stirred to obtain a homogeneous mixture and next put into the oven (for 10 min at 50 °C). The beaker content was poured into the glass molds (100 mm × 80 mm × 2 mm) and polymerized for 24 h at room temperature. The samples were heated at 80 °C for 60 min. Detailed information regarding the reagents and their amounts is presented in Table 1. Figure 2 presents a simple scheme of composite.

## 5. Results

### 5.1. Structural Characterization of Phosphates and Hybrids Using ATR-FT/IR

**Figure 3:**
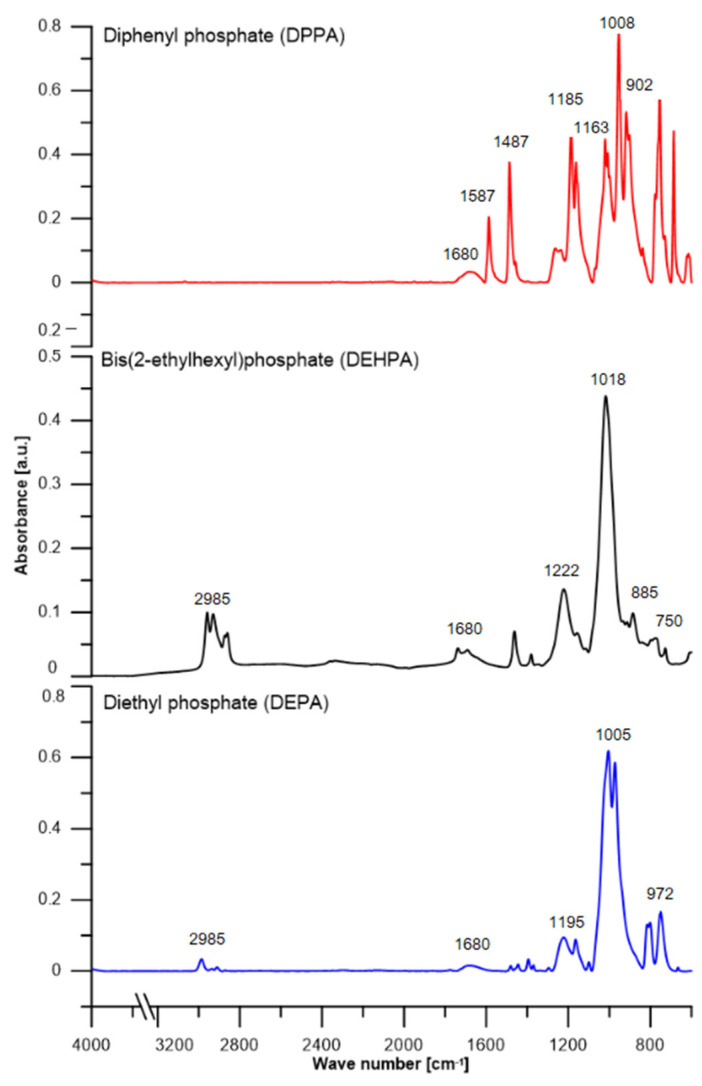
ATR/FT-IR spectra of phosphate samples.

Figure 3 shows ATR-FT/IR spectra of classical absorption bands described in the literature. For the bis(2-ethylhexyl)phosphate (DEHPA) spectrum, a wide band is observed with the maximum at 2958 cm^−1^ from stretching vibrations of carbon-hydrogen bonds presented in the aliphatic chains. Then, numerous absorption bands in the range of 1018 to 885 cm^−1^ are observed, which correspond to the absorption of C-C bonds in the aliphatic chain. Some signals around 750 cm^−1^ are derived from C-O bonds in the structure. The spectra of DEHPA and diethyl phosphate (DEPA) are similar, due to the presence of alkyl substituents in the structure. However, the aliphatic groups in the structure of DEPA give a smaller signal in the range of 2985–2911 cm^−1^, because less energy is absorbed by the bonds in the short carbon chain. In contrast, the spectrum of diphenyl phosphate shows characteristic signals from the aromatic ring: 1487 cm^−1^ from C-H and 1587 cm^−1^ from C=C, as only this one has aromatic rings present in its structure. In comparison with the literature, the following infrared absorption frequencies can be assigned to organophosphoric acids of the type (RO)_2_POOH, as follows: P=O, 1210–1250 cm^–1^; P-O-(H), 1000–1031 cm^–1^; P-O-(C), 987–1042 or 1015–1060 cm^–1^ when R is a methyl group [34]. Importantly, it was observed that the PO band usually appears as a doublet in the infrared spectrum [35]. On this approach, the following assignments can be established for neat DEPA (Table 2): P=O, 1195/1164 cm^–1^; P-O-(C), 1005 cm^–1^; P-O-(H), 972 cm^–1^. Whereas the FTIR absorption spectra of DEHPA and DEPA are comparable, the band associated with the P-O-(H) group that occurs at 972 cm^−1^ in DEPA is not present in the DEHPA spectra. Characteristic band arrangements for phosphorus atom bonds in the DEHPA spectrum are observed for the P-O: 1222/1157 cm^–1^ and P-O-(C), 1017 cm^–1^. Similarly, aromatic phosphoric acid exhibits characteristic bands for the (P=O) and (P-O) stretching vibrations which for DPPA are located at 1185/1163 and 902 cm^–1^, respectively. However, DEHPA, DEPA, and DPPA display broad bands at about 1680 cm^–1^ which are associated with the P-O-H group of the dimeric peak of a hydrogen bond. Furthermore, signals from C-O bonds can be detected in all spectra.

**Figure 4:**
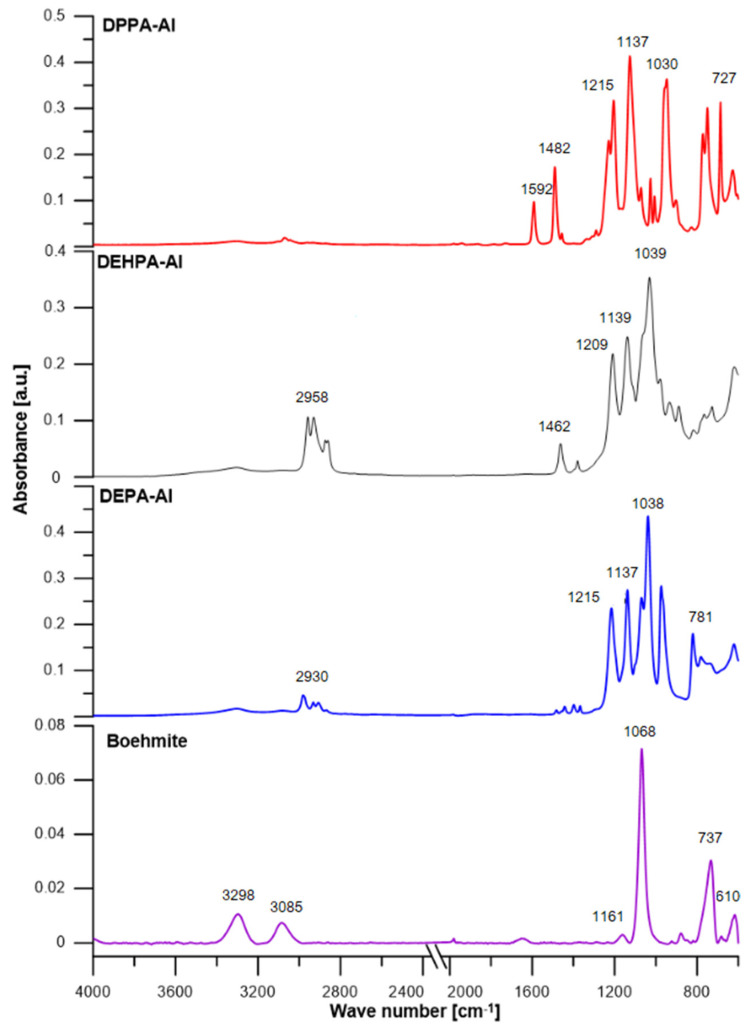
ATR-FT/IR spectra of boehmite and hybrids [cm^−1^].

Figure 4 shows the ATR/FT-IR spectra of boehmite and hybrids. The pure boehmite spectrum shows visible vibration bands in the Al-O-Al bond network (610 cm^−1^), coupled stretching vibrations in the hydroxyl group (3298 and 3085 cm^−1^), and bending vibrations of the Al-OH groups (1161, 1068 cm^−1^ and 737 cm^−1^). The FT-IR absorption spectrum of DEHPA-Al contains three strong signals at 1209, 1139, and 1039 cm^−1^ which can be ascribed to the stretching vibrations of P-O bonds in the bridging units. It isn’t possible to accurately attribute those peaks to specific types of vibrations due to the conjugation of the vibrations of four P-O bonds (two bonds from the Al-O-P-O-Al bridge and two terminal P-O-C bonds) and a lack of details on the geometry of the ligands. By the literature data for FT-IR spectra of well-defined aluminum derivatives of phosphinic acids, the band for symmetric stretching vibrations in Al-O-P-O-Al bridges arises at around 1100 cm^−1^, while the corresponding band for asymmetric vibrations occurs in the region of 1150–1200 cm^−1^ [36]. DPPA, like alkane aluminum phosphate, demonstrates similar bands for (P-O) and (P-O-Al) stretching vibrations at 1215 and 1137 cm^−1^, respectively. In the FT-IR spectra of DPPA-Al, there has been a decrease in the intensity of signals typical of O-H groups in the boehmite structure, together with the presence of a rich spectrum typical of phenyl substituted DPPA anions, with main signals 1292, 1209 cm^−1^ for P-O bonds and a vibration band at 1126 cm^−1^ for P-O-C bonds. Similar signals can be found in the spectrum of DEPA-Al as with other fillers. The stretching vibrations of PO bonds in the bridging sections at 1215, 1137, and 1038 cm^−1^ are responsible for the visible bands. Table 3 also includes the accurate value of the wave numbers.

### 5.2. Morphology of Hybrids and Boehmite

**Figure 5:**
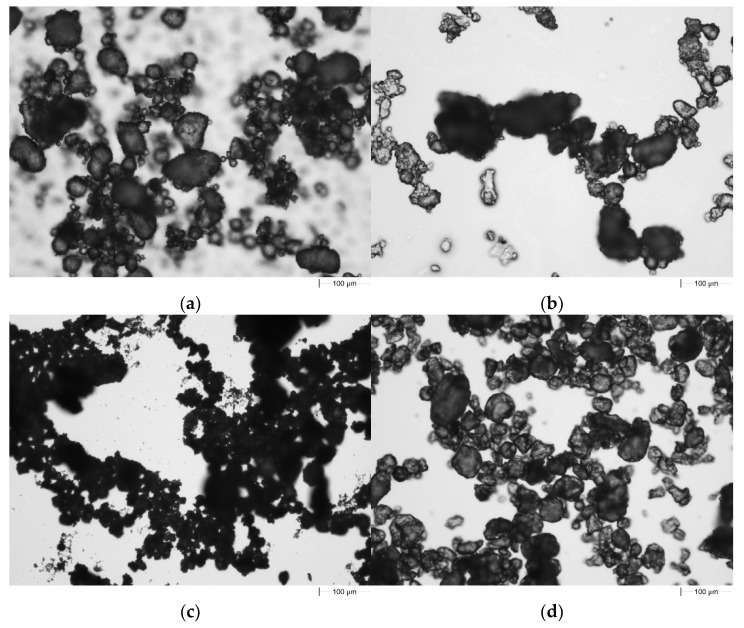
Microscopy observations: (**a**) boehmite, (**b**) DEPA-Al, (**c**) DPPA-Al, (**d**) DEHPA-Al.

Pure boehmite and hybrids were observed by microscopy (Figure 5). The pure boehmite exhibits the most homogenous structure among the fillers. The picture confirmed the boehmite spherical structure characterized by around round particles of different sizes. Hybrid DEPA-Al has elongated particles concentrated in agglomerates. The filler DPPA-Al is characterized by the smallest particles with a diameter lower than 10 µm. The DEPA-Al and DEHPA-Al in the opposition to DPPA-Al have the largest particles with a diameter higher than 50 µm. The samples of modified boehmite are polydisperse. Modified hybrids have a great tendency towards agglomeration which is reflected in lower transparency.

### 5.3. Thermogravimetric Analysis of Hybrids

**Figure 6:**
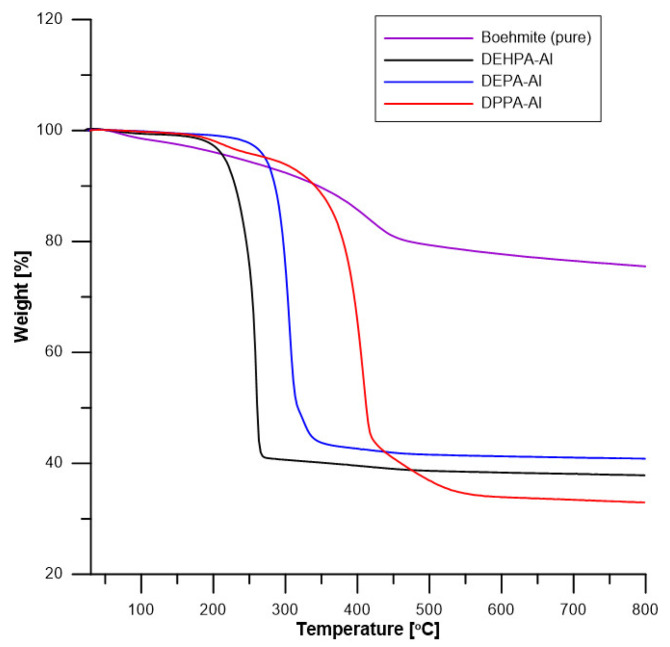
TG curves of boehmite and hybrids (in helium).

**Figure 7:**
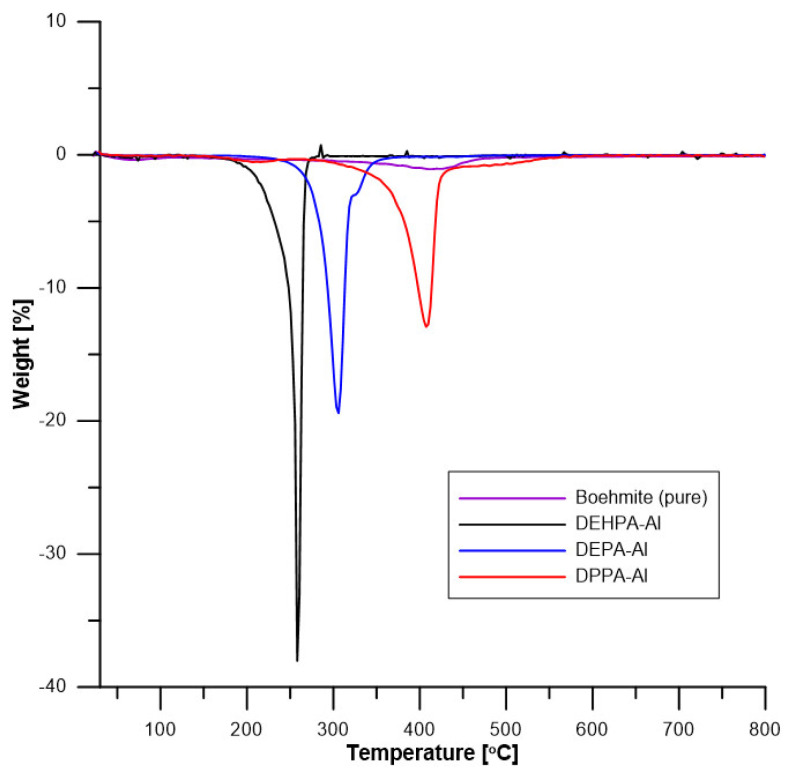
DTG curves of boehmite and hybrids (in helium).

To assess fully the thermal properties of the DEPA-Al, DEHPA-Al, and DPPA-Al samples and pure boehmite, their thermal stability was also determined by thermogravimetric analysis. The data are summarized in Table 4 and Figure 6 and Figure 7.

Pure boehmite loses a small amount of water at low temperatures. The mass loss continues slowly when temperature increases but accelerates at approximately 400 °C. This decomposition corresponds to the endothermic dehydroxylation of boehmite, i.e., the formation of alumina according to the following reaction 2AlO(OH) → Al_2_O_3_ + H_2_O. 

The decomposition of hybrids occurs in one main step but several additional steps can be observed especially for DPPA-Al above 400 °C. The temperature of the main peak changes significantly according to the hybrid. DEHPA-Al decomposes first at around 250 °C. DEPA-Al decomposes at a slightly higher temperature (304 °C). DPPA-Al exhibits the highest thermal stability (its decomposition peak is observed at 408 °C). Thermal stability is very different according to the hybrids.

The residues in the crucible are white. Therefore, no (or negligible) char is formed and residue can be assumed to be only alumina (from boehmite). Pure boehmite let a residue of 76% at 800 °C. Residues from hybrids are 33, 38, and 41 wt% corresponding to boehmite contents of 43, 50, and 54 wt% respectively for DPPA-Al, DEHPA-Al, and DEPA-Al. In other words, around 50% of hybrids are boehmite and 50% are phosphates. The phosphate contents change slightly from one hybrid to another one. It reaches 57 wt% for DPPA-Al and DEHPA-Al and only 46 wt% for DEPA-Al. Such contents correspond to 7.1, 5.3, and 9.2 wt% of P respectively for DPPA-Al, DEHPA-Al, and DEPA-Al (considering the phosphorus content in each phosphate molecule).

### 5.4. Structural Characterization of Composites Using ATR-FT/IR

**Figure 8:**
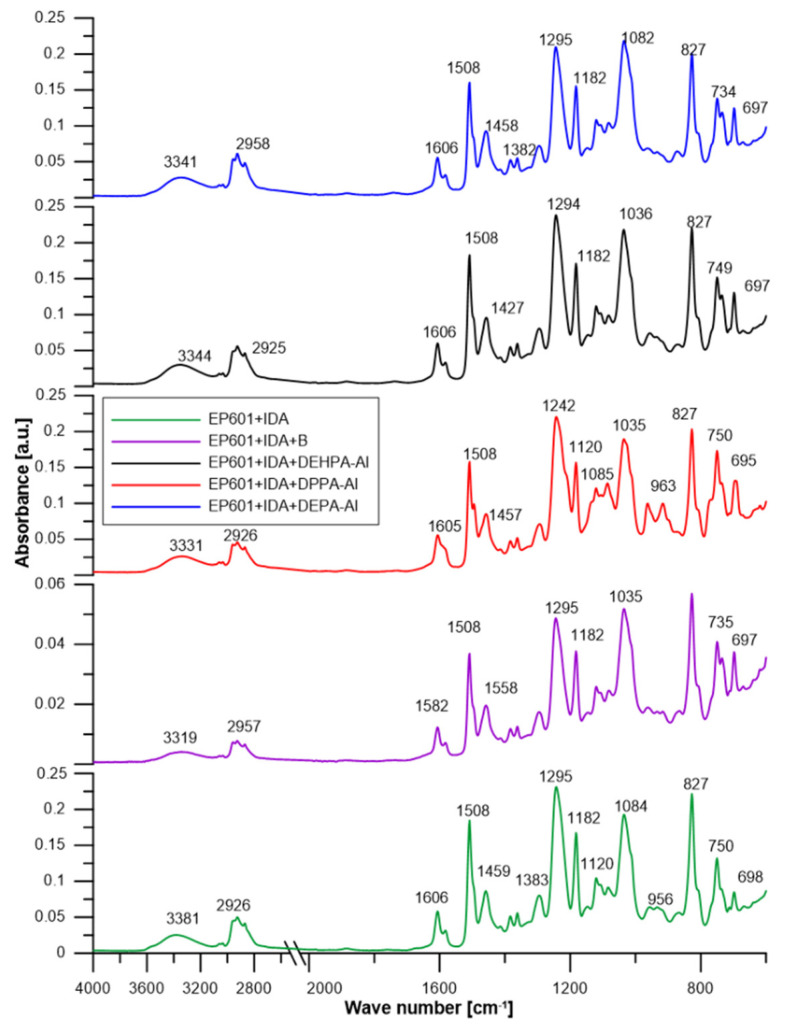
ATR/FT-IR spectra of composites.

Figure 8 presents the spectra of obtained composites. Table 5 additionally includes the precise wavenumber values. In general, the spectra of all obtained composites follow a similar pattern. All composites have a detectable hydroxyl group range from 3319 to 3381 cm^−1^. The presence of -OH groups from Epidian 601 is confirmed by the large absorption band in the spectra. This signal becomes reduced in the modified composites. Stretching vibrations from the C-H aliphatic groups provide another unique absorption band in the 2958–2926 cm^−1^ region. This band can be observed in each composite. Single vibrations of C-H and C=C bonds related to benzene rings and aromatic skeletons can be correlated with single bands ranging from 1606 cm^−1^ to 1449 cm^−1^. They arise from both the aromatic epoxy resin component and diphenyl phosphate addition. Stretching vibrations of the C-O bond could be the cause of the bands of about 1182 cm^−1^. The signal about 800 cm^−1^ was similarly associated with aromatic C-C vibrations (Figure 8). In addition to epoxy resin signals, several particular signals detected in aluminium phosphates are present, confirming the presence of these ATHs in composites.

### 5.5. Pyrolysis Combustion Flow Calorimetry PCFC

**Figure 9:**
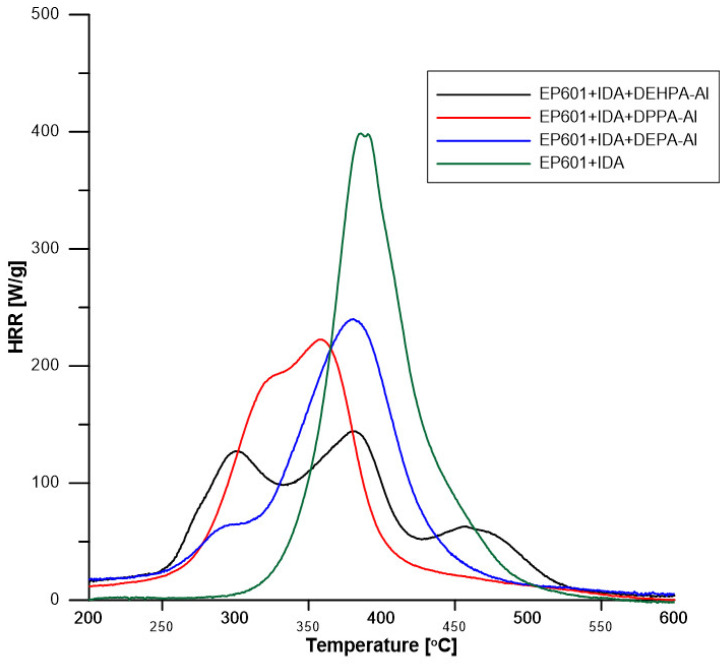
PCFC graphs for obtained composites.

The flammability of the composites was tested at the microscale, using a PCFC—pyrolysis combustion flow calorimeter. Figure 9 and Table 6 present results from PCFC tests.

Pure resin decomposes in one main step with a high pHRR (400 W/g) centered at 396 °C. The decomposition starts at around 300 °C. The residue is negligible and the heat of complete combustion is 28.9 kJ/g. Consequently, THR is 28.3 kJ/g.

Decomposition is much more complex with hybrid with several peaks or shoulders to be observed. In all cases, the decomposition starts earlier (at around 250 °C). The main peak is strongly reduced in comparison to pure resin, i.e., 220–250 W/g and even 150 W/g for the composite containing DEHPA-Al. Residue content is low (around 10 wt%) because the content of boehmite (and then of mineral residue) is low in hybrids and char is not promoted as evidenced in TGA (residues are white). The heat of complete combustion is 22–24 kJ/g, lower than the value measured for epoxy because boehmite releases water, and organic fraction in hybrids releases less heat than epoxy. Indeed, considering that phosphorus is oxidized in P_2_O_5_, the heat of the complete combustion of phosphates ranges from 16.3 kJ/g (for DEPA) to 20 kJ/g (for DEHPA). THR is similar for the three composites containing hybrids, around 21 kJ/g.

### 5.6. Cone Calorimetry

**Figure 10:**
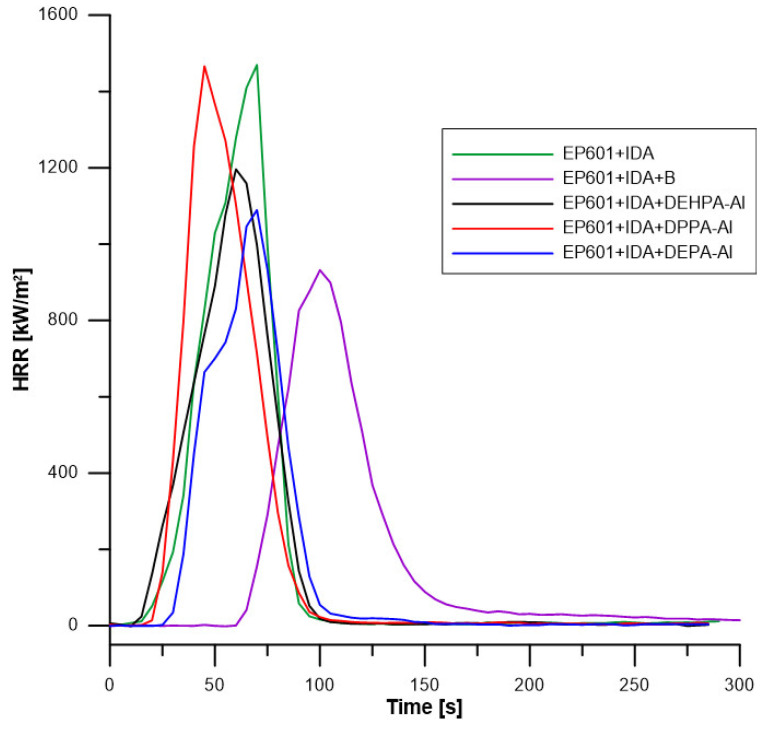
Cone calorimetry graphs for obtained composites.

Figure 10 and Table 7 present results from the cone calorimetry test. Due to their small sizes, all the samples are thermally thin as described by Schartel et Hull [37]. After ignition, the heat release rate increases fast and continuously up to pHRR which is followed by a drastic decrease up to flame out (due to fuel depletion). TTI is 15 s for unfilled matrix and increases significantly for the composite filled with unmodified boehmite up to 64 s. This increase may be due to the release of water which slow downs the surface heating but may be also due to the fast accumulation of boehmites on the surface. In the case of composites filled with hybrids, TTI is much lower and in the same range as the unfilled matrix, probably because phosphorus compounds are released fast and contribute significantly to fuel production. These observations are in agreement with the low thermal stability of these composites observed in PCFC and with the low thermal stability of hybrids in TGA. Especially DEHPA contains a huge amount of carbon and hydrogen and its hybrid has the lowest thermal stability. Its incorporation leads to the lowest TTI and the highest EHC among the composites (close to the value for unfilled resin, −25.5 kJ/g which is typical for epoxy resins) [38].

EHC for other composites tends to decrease because of the water release from boehmite and the slightly lower heat of combustion of organic phosphates. However, this decrease is quite limited (boehmite releases only 15–20% of water and phosphorus compounds do not act as a flame inhibitor—phosphates usually do not act in the gas phase). Indeed, combustion efficiency, calculated as the ratio between EHC and Δh measured in PCFC) is in the range of 0.8–1 in all cases.

Residue content is not significant for the epoxy matrix and increases to 17.7% for the composite containing unmodified boehmite. This residue is slightly higher than the alumina fraction calculated from boehmite decomposition probably due to the formation of a small amount of char. For other composites, the residue content is much lower: Residue contents are close to the values measured in PCFC and correspond quite well to the mineral fraction from boehmite only. Moreover, it must be assumed that phosphates do not promote significantly the charring of the epoxy resin.

PHRR decreases for composites except for DPPA-Al. The decrease is once again quite limited and slightly more significant for the composite containing unmodified boehmite. This may be due to the larger amount of mineral fraction in this composite allowing to form faster a slightly protective residual layer on the top surface of the sample. Such a barrier layer is quite inefficient, especially because the materials are thermally thin: the barrier effect is much more protective for thermally thick materials.

Finally, the total smoke release is reduced in presence of unmodified boehmite. This is directly related to the HRR reduction. Indeed, smoke depends not only on the nature of burnt material but also on the heat release rate [39]. However, phosphates lead to an increase in TSR which reaches the same values as the unfilled resin.

**Figure 11:**
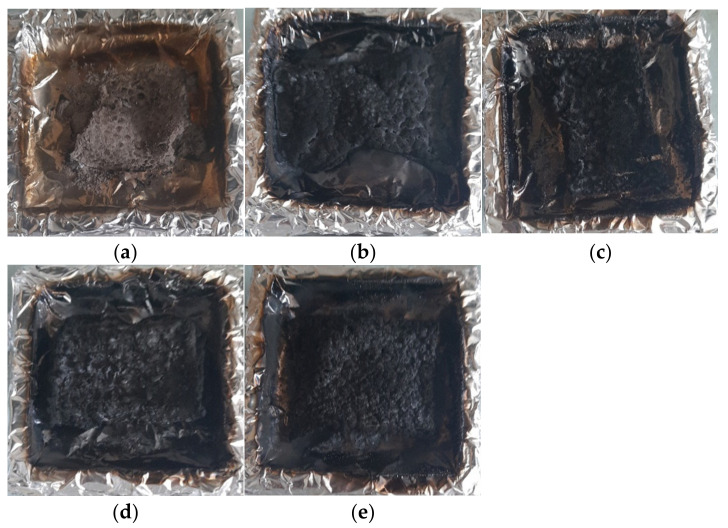
Photos of residue from cone calorimetry tests: (**a**) EP601 + IDA, (**b**) EP601 + IDA + B, (**c**) EP601 + IDA + DEHPA-Al, (**d**) EP601 + IDA + DPPA-Al, (**e**) EP601 + IDA + DEPA-Al.

Figure 11 shows the aspect of residues after the cone calorimeter: whichever the composite, no cohesive and thick layer can be observed confirming that barrier effect nor significant char promotion occurs.

Ferry et al. described Polyamide 11-nanoboehmite composites for thermal degradation and flammability properties. The presence of nanoboehmite reduces the time to ignition. PHRR was slightly affected at low boehmite content (10%), whereas a significant decrease was observed at higher content (30%). The 10% nanoboehmite composition behaves very similarly to pure PA11, whereas the 30% nanofiller composition exhibits significantly lower HRR peaks [40]. Laachachi et al. compared the role of boehmite (AlOOH) and alumina (Al_2_O_3_) in the thermal stability and flame-retardant behavior of poly(methyl methacrylate) nanocomposites. Cone calorimeter measurements showed that the peak of heat release rate is lowered in the presence of AlOOH or Al_2_O_3_ in comparison to pure PMMA and that this decrease is higher when the filler content increases. They concluded that the prepared nanocomposites showed high thermal stability and higher flame retardancy in comparison with pure PMMA. A greater reduction in pHRR (44%) was recorded with PMMA–20%AlOOH and PMMA–15%Al_2_O_3_ samples [41]. Although, Pawlowski et al. have tested blends based on PC/ABS (Polycarbonate/Acrylonitrile-Butadiene-Styrene) flame retarded with boehmite and aryl phosphates. They stated that adding 5 wt.% AlOOH or 5 wt.% of AlOOH and 12.5 wt% of BDP to blend results in significantly enhanced barrier effects of the fire residue during burning. Nevertheless, these authors consider that the combination of phosphates and boehmite must be analyzed “with caution and circumspection”. Indeed, they concluded that phosphorus reacts with boehmite to form AlPO_4_, preventing its action in the condensed phase (charring promotion), while its action in the gas phase is not disturbed [42].

### 5.7. Hardness Test

**Figure 12:**
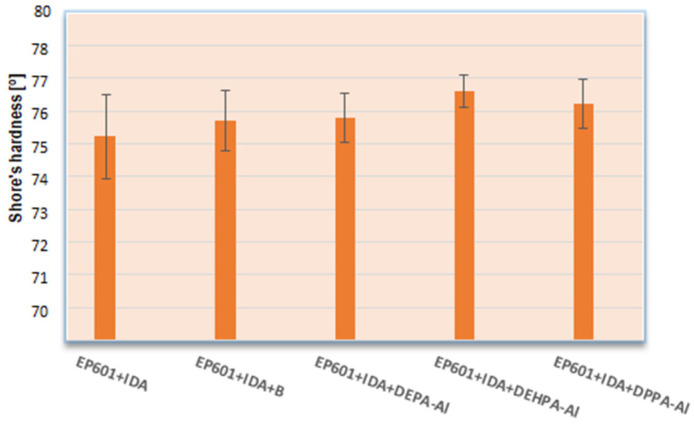
Shore’s hardness of composites.

The Shore hardness values of the composites is presented on Figure 12. The pure composite without addition had the lowest Shore hardness (76.2° Sh). Analyzing the obtained results one can conclude that the addition of modified boehmites (APH) raises slightly the composite’s hardness by about 0.5–1.4° Sh. The little effect of fire retardant compound addition on the hardness of the composites is observed but it is also worth noting that pure boehmite also hardened the material. The range of this parameter for the materials was 0.6–1.4° Sh units in the D scale. According to the hardness tests it can be assumed that the modification type slightly contributes to the changing hardness of the obtained materials.

## 6. Conclusions

Combining phosphates and mineral fillers is often a good solution to improve the flame retardancy of polymers. In this study, by reacting aluminum oxide hydroxide (boehmite) with alkyl and aryl phosphoric acids, new inorganic hybrid materials were obtained. Initially, two phosphorus organic derivatives were synthesized and their structure was confirmed by the NMR method. Then, the boehmite was successfully modified by diphenyl phosphate, diethyl phosphate, and bis(2-ethylhexyl)phosphate. The epoxy composites with 17 wt% of raw boehmite or hybrids were prepared to assess their flammability.

The structure of obtained hybrids and composites were confirmed by infrared spectroscopic analyzes. Microscopic observations showed the sphericity of boehmite particles but the samples of modified boehmite are polydisperse. Thermogravimetry analysis informed that thermal stability is very different according to the hybrids. The decomposition of hybrids occurs in one main step but several additional steps can be observed especially for DPPA-Al above 400 °C. DEHPA-Al decomposes first at around 250 °C. DEPA-Al decomposes at a slightly higher temperature (304 °C). DPPA-Al exhibits the highest thermal stability its decomposition peak is observed at 408 °C. Nevertheless, in obtained composites flame retardant performances were not enhanced in presence of hybrids. While unmodified boehmite allows a slight reduction in pHRR and strongly delays the ignition, the combination of boehmite and phosphates in hybrids is detrimental to the fire behavior. First, phosphates do not act efficiently in the gas phase or in the condensed phase. Second, they contain a large amount of carbon and hydrogen contributing to feed the flame. Finally, hybrids contain a lower amount of minerals and then the formation of a protective layer is delayed. To conclude, the flame-retardant effect of boehmite and its hybrids on rigid, polymeric epoxy-derived composites has been studied and confirmed for the first time.

## Figures and Tables

**Table 1:** Amounts of chemicals *.

Name of Sample	EP601 [g]	IDA [g]	B [g]	DEPA-Al [g]	DEHPA-Al [g]	DPPA-Al [g]
EP601 + IDA	12	6	0	0	0	0
EP601 + IDA + B	12	6	3.6	0	0	0
EP601 + IDA + DEPA-Al	12	6	0	3.6	0	0
EP601 + IDA + DEHPA-Al	12	6	0	0	3.6	0
EP601 + IDA + DPPA-Al	12	6	0	0	0	3.6

* Abbreviations: B–pure boehmite; DEPA-Al–boehmite-diethyl phosphate hybrid; DPPA-Al–boehmite-diphenyl phosphate hybrid; DEHPA-Al–boehmite-bis(2-ethylhexyl)phosphate hybrid.

**Table 2:** Characteristic bands of ATR-FT/IR spectra of phosphates [cm^−1^].

Product	C-H Aliph.	C-H Arom.	C-C	C-O	P=O	P-O-(C)	P-OH
DEPA	2985 2911	-	1445 1370	802 751	1195 1164	1005	972
DEHPA	2958 2930	-	1462 1380	885 771 727	1222 1157	1017	-
DPPA	-	1486	-	954 755 687	1185 1163	1008	902

**Table 3:** Characteristic bands of ATR-FT/IR spectra of hybrids [cm^−1^].

Sample	C-H Aliph.	C-H Arom.	C=C Arom.	C-C	C-O	P-O	P-O-Al	P-O-(C)
DEPA-Al	2981 2931	-	-	1482 1398	781 621	1215	1137	1038
DEHPA-Al	2958 2930	-	-	1462 1379	727 619	1209	1139	1030
DPPA-Al	-	1492	1592	-	947 772 627	1292	1209	1126

**Table 4:** TG data for hybrids in helium.

Sample	Initial Decomposition Temperature [°C]	Maximum Weight Loss Temperature [°C].	Maximum Decomposition Rate [%/min]	Residue [%]	Mineral Fraction [%]	Phosphorus Content (wt%)
DEPA-Al	280	304	35	41	54	9.2
DEHPA-Al	224	258	39	38	50	5.3
DPPA-Al	358	408	27	33	43	7.1
Boehmite	86	476	25	76	100	0

**Table 5:** TG data for hybrids in helium [cm^−1^].

Polymer	C-H Aliph.	C-H Arom.	C=C	C-C	C-O	-OH
EP601 + IDA	2926 2870 1383	1449 1084	1606 1508 698	1182 1120 956	1295 827 750	3381
EP601 + IDA + B	2957	1458 1035	1582 1508 697	1182	1295 735	3319
EP601 + IDA + DEPA-Al	2958	1458	1606 1508 697	1382 1182 1082	1295 827 734	3341
EP601 + IDA + DEHPA-Al	2925	1457 1036	1606 1508 697	1182	1294 827 749	3344
EP601 + IDA + DPPA-Al	2926	1457 1035	1605 1500 695	1120 1085 963	1242 827 750	3331

**Table 6:** Results for PCFC tests.

Polymer	pHRR [W/g]	T_pHRR_ [°C]	THR [kJ/g]	Residue Fraction	Δh [kJ/g]
EP601 + IDA	396	396	28.3	28.3	28.9
EP601 + IDA + DEPA-Al	245	387	23.7	23.7	23.3
EP601 + IDA + DEHPA-Al	148	382	20.8	20.8	22.3
EP601 + IDA + DPPA-Al	223	365	21.3	21.3	24.3

pHRR–peak of heat release rate; T_pHRR_–temperature of heat release rate; THR–total heat release.

**Table 7:** Results from cone calorimetry.

Sample Name	Time-to-Ignition [s]	pHRR [kW/m^2^]	THR [MJ/m^2^]	THR [kJ/g]	EHC [MJ/kg]	Char Content Residue [%]	Total Smoke Release [m^2^/m^2^]
EP601 + IDA	15	1469	53.2	25.65	25.5	1.7	1546
EP601 + IDA + B	64	932	46.7	17.63	21.9	17.7	1290
EP601 + IDA + DEPA-Al	29	1089	43.0	19.03	21.2	12.5	1668
EP601 + IDA + DEHPA-Al	14	1196	50.2	21.18	24.8	11.6	1594
EP601 + IDA + B + DPPA-Al	21	1466	54.0	22.09	21.9	10.1	1521

Time—time to ignition; pHRR–peak of heat release rate; HRR—heat release rate; THR—total heat release; EHC—effective heat of combustion.

## Data Availability

Not applicable.
